# Human tau increases amyloid β plaque size but not amyloid β‐mediated synapse loss in a novel mouse model of Alzheimer's disease

**DOI:** 10.1111/ejn.13442

**Published:** 2016-11-12

**Authors:** Rosemary J. Jackson, Nikita Rudinskiy, Abigail G. Herrmann, Shaun Croft, JeeSoo Monica Kim, Veselina Petrova, Juan Jose Ramos‐Rodriguez, Rose Pitstick, Susanne Wegmann, Monica Garcia‐Alloza, George A. Carlson, Bradley T. Hyman, Tara L. Spires‐Jones

**Affiliations:** ^1^ Centre for Cognitive and Neural Systems and Centre for Dementia PreventionThe University of Edinburgh1 George SquareEdinburghEH8 9JZUK; ^2^ Massachusetts General Hospital and Harvard Medical SchoolCharlestownMAUSA; ^3^ University of CadizCadizSpain; ^4^ McLaughlin Research InstituteGreat FallsMTUSA

**Keywords:** Alzheimer, amyloid beta, plaque, synapse, tau

## Abstract

Alzheimer's disease is characterized by the presence of aggregates of amyloid beta (Aβ) in senile plaques and tau in neurofibrillary tangles, as well as marked neuron and synapse loss. Of these pathological changes, synapse loss correlates most strongly with cognitive decline. Synapse loss occurs prominently around plaques due to accumulations of oligomeric Aβ. Recent evidence suggests that tau may also play a role in synapse loss but the interactions of Aβ and tau in synapse loss remain to be determined. In this study, we generated a novel transgenic mouse line, the APP/PS1/rTg21221 line, by crossing APP/PS1 mice, which develop Aβ‐plaques and synapse loss, with rTg21221 mice, which overexpress wild‐type human tau. When compared to the APP/PS1 mice without human tau, the cross‐sectional area of ThioS+ dense core plaques was increased by ~50%. Along with increased plaque size, we observed an increase in plaque‐associated dystrophic neurites containing misfolded tau, but there was no exacerbation of neurite curvature or local neuron loss around plaques. Array tomography analysis similarly revealed no worsening of synapse loss around plaques, and no change in the accumulation of Aβ at synapses. Together, these results indicate that adding human wild‐type tau exacerbates plaque pathology and neurite deformation but does not exacerbate plaque‐associated synapse loss.

## Introduction

Alzheimer's disease (AD) is a progressive neurodegenerative disease that is the most common cause of dementia in the elderly. It is characterized neuropathologically by the aggregation of amyloid beta (Aβ) into senile plaques and tau into neurofibrillary tangles (NFTs), as well as by pronounced synapse loss, neuron loss and gliosis (Duyckaerts *et al*., 2009).

The amyloid cascade hypothesis of disease pathogenesis posits that AD is initiated by the accumulation of Aβ, which then leads to glial‐, neuronal‐ and tau‐related neuropathological hallmarks (Hardy & Higgins, 1992). This hypothesis is strongly supported by the genetics of familial early onset AD, which is caused by mutations in the proteins that are essential for the generation of Aβ: amyloid precursor protein (APP), presenilin‐1 (PS1) and presenilin‐2 (PS2; Tanzi, 2012). The accumulation of Aβ plaques correlates less well with cognitive decline in AD than NFTs (Ingelsson *et al*., 2004), although mutations in the tau gene, *Mapt,* cause fronto‐temporal dementia (FTD) but not AD (Hutton, 2000). However, neither plaques nor tangles correlate as well with cognitive decline as synapse loss, emphasizing the important role of synapse toxicity for pathogenesis and disease progression in AD (Spires‐Jones & Hyman, 2014).

Animal models and human studies have shown that soluble oligomers of both Aβ‐ and FTD‐associated mutant tau contribute to synapse loss (Walsh *et al*., 2002; Shankar *et al*., 2008; Koffie *et al*., 2009, 2012; Kopeikina *et al*., 2011; Lasagna‐Reeves *et al*., 2011; Bilousova *et al*., 2016). When expressed in the same animal model, these small pathological molecules can affect the distribution and pathology of each other (Oddo *et al*., 2003; Pooler *et al*., 2015). Modulation of one of the pathologies often affects the other in these models, strongly indicating that these processes are interconnected (Oddo *et al*., 2006; Castillo‐Carranza *et al*., 2015) but the molecular pathways linking Aβ, tau, and synapse degeneration remain largely unknown.

There is strong evidence that tau is necessary for Aβ‐mediated synaptic pathology in animal models of plaque deposition. Genetically reducing endogenous tau is protective against synaptic phenotypes including LTP deficits, seizures, and axonal transport deficits (Roberson *et al*., 2007, 2011; Shipton *et al*., 2011; Vossel *et al*., 2015). However, there are alternative hypotheses stipulating that tau and Aβ pathologies start independently but then act synergistically to cause synapse loss and cognitive decline (Small & Duff, 2008).

In this study, we examine Aβ‐plaque load and synapse loss in the presence of human tau. We generate a mouse model (APP/PS1/rTg21221) of early AD, in which mutant human APP, PS1, and wild‐type human tau are co‐expressed. This novel mouse line allows analysis of interactions of human tau and Aβ in a mammalian brain with age‐related pathology (which cannot be fully recapitulated *in vitro*). APP/PS1 mice expressing both mutant APP and PS1 (but no human tau) show plaque deposition at 4–6 months of age (Jankowsky *et al*., 2004). Cognitive impairments, synapse loss around plaques (Koffie *et al*., 2009) and disruption of Ca2+ regulation in dendritic spines are well‐established phenotypes of this line (Wu *et al*., 2010). In contrast, rTg21221 mice that overexpress wild‐type human tau (hTau) show only slight behavioral phenotypes and tau hyperphosphorylation, no tau aggregation into NFTs, and no synapse loss (Hoover *et al*., 2010). By crossing these two lines, we tested the synergy between Aβ and tau, how tau contributes to Aβ‐related pathology, and how Aβ effects non‐mutant tau.

We hypothesized that increasing the amount of tau in a mouse model of AD would worsen synapse loss. However, surprisingly, we found that while over‐expressing wild‐type human tau increases Aβ‐plaque size and dystrophic neurite number, it does not exacerbate Aβ‐mediated synapse loss, neuron loss or gliosis.

## Materials and methods

### Generation of App/PS1/rTg21221 mice

B6.C3 APP/PS1 mice (stock #004462, Jackson Laboratories Bar Harbor, ME), which express APP^swe^ and PS1^Δexon9^ (Jankowsky *et al*., 2004), were crossed with B6.129‐Tg(CK‐tTA) mice that express the tet transactivator, CK‐tTA, under the control of the calcium calmodulin kinase 2 alpha (CamK2α) promoter. Offspring positive for both the APP/PS1 and CK‐tTA transgene were then crossed to transgene homozygous Tg(tetO‐HuTau_wt_) 21221 mice (Hoover *et al*., 2010). Expression of human wild‐type human 4‐repeat tau in rTg21221 mice is under the control of a dox‐off tetracycline transactivator responsive promoter. Mice positive for the APP/PS1, CK‐tTA and rTg21221 transgenes (APP/PS1/rTg21221) overexpress APP^swe^ and PS1^Δexon9^ as well as wild‐type human tau in the forebrain.

Mice used were 8–10 months old and of mixed sex. Animals were group housed, and had *ad libitum* access to food and water. Animals were killed with CO_2_ and brains collected. Brain hemispheres were fixed for 48 h in 4% paraformaldehyde (PFA) in PBS, cryoprotected in 15% glycerol in PBS, then frozen in dry ice and sectioned into 50 μm thick coronal sections using a freezing microtome (Leica 2010R). From the other hemisphere, small samples of somatosensory cortex (1 × 1 × 5 mm^3^) were dissected freshly from each brain for array tomography, and the remainder brain tissue was flash‐frozen at −80 °C for biochemical analyses. The number of animals used in each experiment can be found in the figure legend for that experiment. All animal experiments conformed to national and institutional guidelines including the Animals [Scientific Procedures Act] 1986 (UK), and the Council Directive 2010/63EU of the European Parliament and the Council of 22 September 2010 on the protection of animals used for scientific purposes, and had full IACUC and Home Office ethical approval.

### Synaptoneurosome preparation

Synaptoneurosomes and crude homogenate were prepared as described previously (Tai *et al*., 2012). In brief, < 100 mg of frozen murine frontal cortex was homogenized in 700 μL of ice‐cold buffer A (25 mmol/L HEPES pH 7.5, 120 mmol/L NaCl, 5 mmol/L KCl, 1 mmol/L MgCl2 and 2 mmol/L CaCl2), supplemented with 2 mmol/L di‐thiothreitol, protease inhibitors (Roche complete mini) and phosphatase inhibitors. The homogenate was passed through two layers of 80‐μm nylon filters (Millipore, Watford, UK), and a 200 μL aliquot of the filtered homogenate was saved. The saved aliquot was mixed with 200 μL water and 70 μL 10% SDS, to prepare the crude homogenate.

To prepare synaptoneurosomes, the remainder of the homogenate was passed through a 5‐μm Durapor membrane filter (Millipore) to remove large organelles and nuclei and centrifuged at 1000 ***g*** for 5 min. The non‐synaptic supernatant containing cytoplasmic proteins was removed, and the pellet was washed once with buffer A and centrifuged again, yielding the synaptoneurosome pellet. The synaptoneurosome pellet was suspended in 400 μL of Buffer B (50 mmol/L Tris [pH 7.5], 1.5% SDS, and 2 mmol/L DTT) and boiled for 5 min. Protein concentrations were determined using a BSA assay (Thermo Fisher, Renfrew, UK).

### Western blotting

Five microgram of protein from either isolated synaptoneurosomes or crude homogenate was loaded onto NuPAGE 4–12% Bis‐Tris precast polyacrylamide 15 well gels (Invitrogen, Paisley, UK) along with molecular weight marker (Li‐Cor, Cambridge, UK). Proteins were electro‐transferred to nitrocellulose membrane (Bio‐Rad, Hemel Hempstead, UK). Membranes probed with the following primary antibodies: Aβ(82E1,IBL,1 : 100), Tau13 (MMS‐520R‐500, Covance, 1 : 2000), β‐actin (ab8226, Abcam, 1 : 2000), Synaptophysin (AB8049, Abcam, 1 : 10 000), α‐tubulin (ab4074, Abcam, 1 : 1000), GFAP (0334, DakoCytomation, 1 : 500), GAPDH (ab8245, Abcam, 1 : 2000). Proteins were visualized on an odyssey infrared system using the appropriate 680 and 800 IR dye secondary antibodies (1 : 50 000, LI‐COR Biosciences) and were analyzed using odyssey software (LI‐COR Biosciences).

### ELISA

Aβ42 concentration was quantified, in isolated synaptoneurosomes, using a colorimetric Aβ42 ELISA kit (Wako, Japan) as previously described with minor modifications (Ramos‐Rodriguez *et al*., 2016). Briefly, 5 μL of synaptoneurosomes were diluted in 50 μL of lysis buffer with inhibitor cocktail (Thermo Scientific Pierce, Spain). Samples were loaded and standard curves were completed with human Aβ42 provided in the kit. Absorbance was measured spectrophotometrically at 450 nm (MQX200R2, Biotekinstruments, Burlington VT, USA) and data were expressed as pMol Aβ42/mg synaptoneurosome protein.

### Immunohistochemical analyses of plaque load, neuron loss, gliosis and neurite damage

To quantify plaque‐associated neuron loss, a series of every 10th coronal section through the brain hemisphere was stained with 0.05% Thioflavin‐S (ThioS) in 50% ethanol for 8 min to label dense‐core plaques and NFTs and washed in 80% ethanol for 30 s. Sections were then permeabilized for 10 min in 0.1% Triton X‐100 in TBS, washed twice for 5 min in TBS, then incubated in NeuroTrace red fluorescent Nissl stain (1 : 500; Molecular Probes, Inc.) for 1 h at room temperature to label neurons. Sections were mounted onto superfrost plus slides using Immuno‐Mount mounting media and a cover slip was placed on top. Another series of sections were stained with primary antibody against glial‐fibrillary acidic protein (GFAP; 1 : 1000, Dako Cytomation 0334) to stain for reactive astrocytes and the Alexa Fluor conjugated secondary antibody donkey anti‐rabbit 647 (1 : 200; Life Technologies, Carlsbad, CA). Sections were counterstained with ThioS and mounted onto slides as described above.

Low‐resolution tile scan images of every other section in the series (coronal sections 1 mm apart) were taken at 5× magnification with an epifluorescence microscope (Zeiss Axio Imager Z2; Carl Zeiss, Ltd., Cambridge, UK). Cortical thickness was measured at three equi‐distant points on each section. The cortex was outlined and 6–10 plaques in each section were randomly chosen and imaged at 63× magnification (1.4 NA plan apochromat objective) using the axiovision rel. 4.8.2 software to take a z‐stack through each section at every 3 μm thickness. Neuronal numbers in the vicinity of dense‐core plaques (in a 30 × 30 × 50 μm volume) and far from plaques (at least 100 μm in a 30 × 30 × 50 μm volume in the same cortical layer) were obtained by stereological sampling using steroinvestigator software. Plaque burden (the percentage of cortex occupied by plaques) and individual plaque area were measured in image j. For astrocyte counts, the same sampling scheme was used on GFAP stained sections to choose plaques on low‐resolution images and take high‐resolution images of 6–10 plaques per section on every 20th section. GFAP‐positive astrocytes around dense‐core plaques were counted in a 30‐μm radius circle from the edge of the plaque.

To label dystrophic neurites and axons, Alz50 (kind gift of Peter Davies) and Smi312 (ab24574, Abcam) were used at concentrations of 1 : 1000 and 1 : 5000, respectively. Alz50‐positive neurites within the area of the ThioS‐positive plaques were counted. Neurite curvature was calculated by measuring the length of each axon segment and dividing it by the end‐to‐end distance of the segment. Neurite distance from a plaque was calculated by taking an average of the distance to the plaque from each end and the middle of the axon segment measured. Axons were only measured if they could be followed up for more than 20 μm and in total, 339 axons from eight mice were measured and an average was taken for each mouse.

### Array tomography

Brain tissue from somatosensory cortex was prepared for array tomography as described previously (Micheva & Smith, 2007; Kay *et al*., 2013). In brief, tissue from five APP/PS1/rTg21221 and six APP/PS1 mice was embedded in acrylic resin and cut into ribbons of 70 nm sections that were collected on gelatin‐coated glass coverslips. The ribbons were then stained with antibodies and imaged along the ribbon. The ribbons were then stripped (0.2 m NaOH, 0.02%SDS in dH_2_O) and reprobed with a second set of antibodies and images were taken in the same location as those on day 1. Primary antibodies on day 1 were 1C22 [1 : 50, kind gift of Dominic Walsh (Yang *et al*., 2015)], rabbit anti‐synapsin‐1 (1 : 100, AB1543P, Millipore) and goat anti‐PSD95 (1 : 50, ab12093, Abcam). Primary antibodies used on day 2 were AW7 (1 : 1000, kind gift of Dominic Walsh), mouse anti‐Tau13 (1 : 50, MMS‐520R‐500, Covance) and goat anti‐PSD95 (1 : 50, ab12093, Abcam). 1C22 recognizes conformer specific oligomeric Aβ and was raised in a mouse and AW7 recognizes total Aβ and was raised in a rabbit. Secondary antibodies were purchased from Invitrogen and were used at 1 : 50. Alexa Fluor conjugated secondary antibodies used on day 1 were donkey anti‐mouse 488 (A21202), donkey anti‐rabbit 594 (A21207) and donkey anti‐goat 647 (A21447). Secondary antibodies used on day 2 were donkey anti‐mouse 488 (A21202), donkey anti‐rabbit 647 (A31573) and donkey anti‐goat 594 (A11058).

Images from each section in the ribbon were complied to create a 3D stack and aligned using imagej multistackreg macros (Thevenaz *et al*., 1998). Regions of interest (10 × 10 μm) were selected near plaques (< 20 μm) and far from plaques (> 40 μm). Images were thresholded in imagej/fiji (Schindelin *et al*., 2012) and custom matlab macros were used to remove single slice punctate, count synaptic punctuate and assess co‐localization with 1C22 (all custom analysis macros will be freely available along with data spreadsheets supporting this manuscript at http://dx.doi.org/10.7488/ds/1507).

### Statistics

In these experiments, we compare APP/PS1 mice to APP/PS1/rTg21221 mice with the experimental unit being a single animal. Numbers of animals in each experiment are shown in figure legends. For each parameter, a mean or median (depending on normality) was calculated for each animal, and then the group mean or median was calculated. The null hypothesis was no difference between APP/PS1 and APP/PS1/rTg21221 mice for each measured parameter. Statistical analysis on the data obtained was performed using spss software (version 21 IBM Armonk, New York, USA). Each data set was individually tested for normal distribution using the Shapiro–Wilk normality test. When data were normally distributed such as the astrocytes quantification around plaques, anova or Students *T*‐test was used to test for difference between the means for each individual animal data across the experimental conditions. Tukey's *post hoc* multiple comparisons test was also applied. When data were not normally distributed, such as the neuronal and microglial counts as well as the quantification of the western blot data, a non‐parametric Kruskal–Wallis test was used to test for significant difference between the medians for each individual animal across the experimental groups. All statistics were carried out at 95% confidence intervals, therefore a significant threshold of *P* < 0.05 was used in all analyses; the number of mice used in each experiment can be found in the figure legends.

## Results

### Effects of hTau overexpression on pathology in APP/PS1 mice

**Figure 1:**
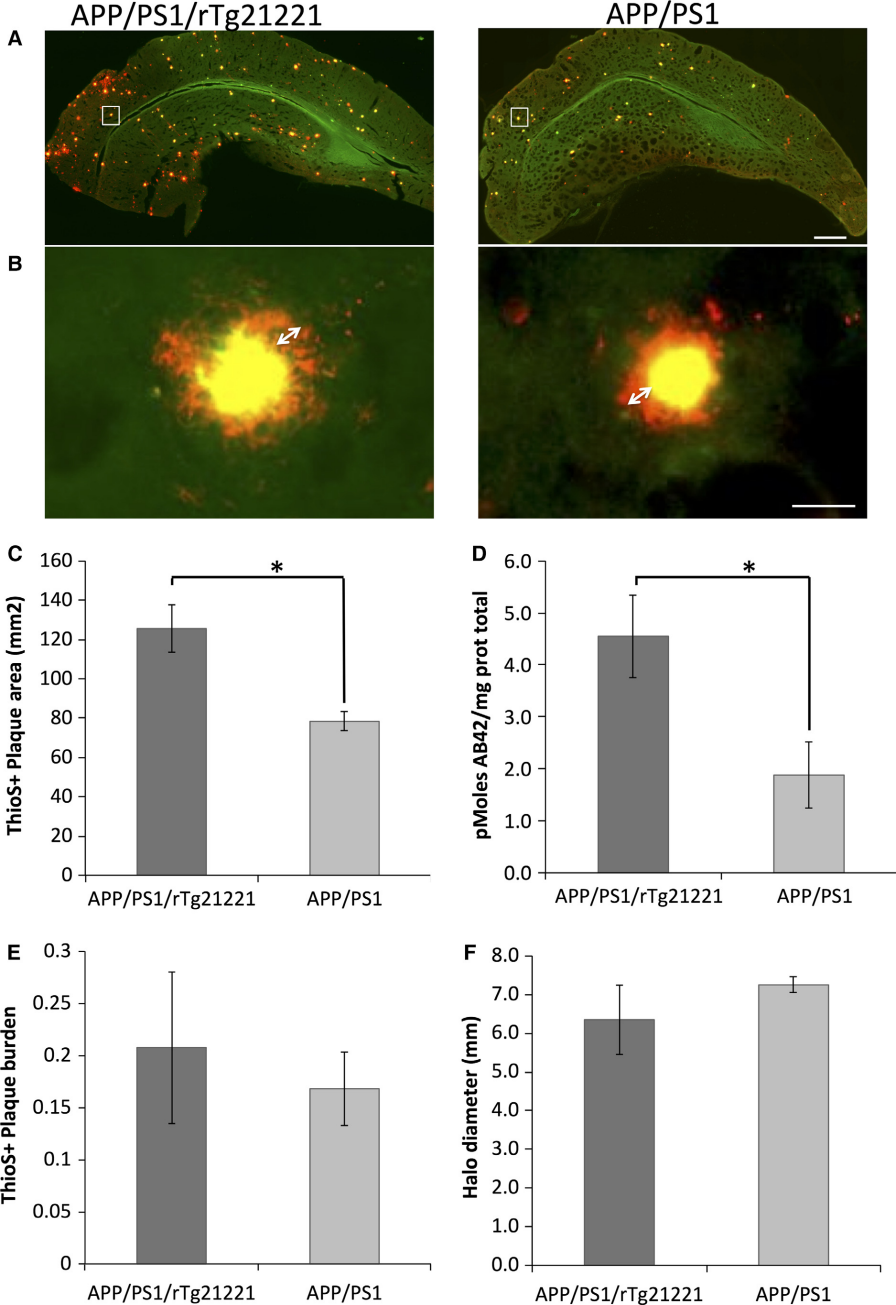
Overexpression of human tau increases the cross‐sectional area of ThioS‐positive plaques but not the overall plaque burden. Representative images of dense Aβ‐plaques (ThioS, yellow) and total Aβ (AW7, red) in brain sections were used to measure plaque characteristics (A). Higher resolution images demonstrate the halo of soluble Aβ (white arrows) surrounding dense plaques in APP/PS1/rTg21221 mice and APP/PS1 mice (B). The mean cross‐sectional area of individual ThioS‐positive plaques increased in APP/PS1/rTg21221 compared to APP/PS1 mice (*t‐test, df = 6, *P* = 0.03) (C). ELISA on crude brain homogenates showed a significant increase (*t‐test df = 8.673, *P* = 0.023) in the amount of Aβ42 in APP/PS1/rTg21221 mice (D). The percentage area of cortex occupied by plaques (plaque burden) was unchanged (E) as was the thickness of the soluble Aβ halo around dense plaques (F). APP/PS1/rTg21221 *n* = 5, APP/PS1n = 3, scale bars represent 500 μm in (A) and 20 μm in (B). [Colour figure can be viewed at wileyonlinelibrary.com].

Raw analysed data from this manuscript and custom analysis macros used in analysis are available at http://dx.doi.org/10.7488/ds/1507. APP/PS1 mice begin to develop amyloid plaques at around 4–6 months of age (Garcia‐Alloza *et al*., 2006b). To determine whether hTau overexpression affects plaque deposition in these 8–10‐month‐old animals, Thioflavin‐S (ThioS) was used to stain dense plaques, and AW7 was used to immunolabel all Aβ depositions. Cortical ThioS‐positive plaque burden was unchanged with hTau overexpression (0.21 ± 0.16% in APP/PS1/rTg21221 mice; 0.17 ± 0.06% in APP/PS1 mice without hTau), as was the burden of total Aβ−plaques immunostained with AW7 (0.55 ± 0.42% in APP/PS1/rTg21221 mice; 0.39 ± 0.05% in APP/PS1 mice, Fig. 1E). The variability in plaque burden in the mice overexpressing human tau was very high, and we did observe a significant increase (df = 6, *P* = 0.03, *t*‐test) in the size (the average cross‐sectional area of plaques) of ThioS‐positive plaques in APP/PS1/rTg21221 (Fig. 1C). There was no difference in AW7‐positive plaque size or the diameter of the halo of Aβ around dense plaques. Consistent with larger dense plaques detected with histology, we observed an increase in Aβ42 levels in brain homogenates by ELISA in APP/PS1/rTg21221 (Fig. 1D; 4.6 pmol Aβ42/mg protein) compared to APP/PS1 mice (2.1 pmol Aβ42/mg protein, df = 8.673 *P* = 0.023 Kruskal–Wallis test).

**Figure 2:**
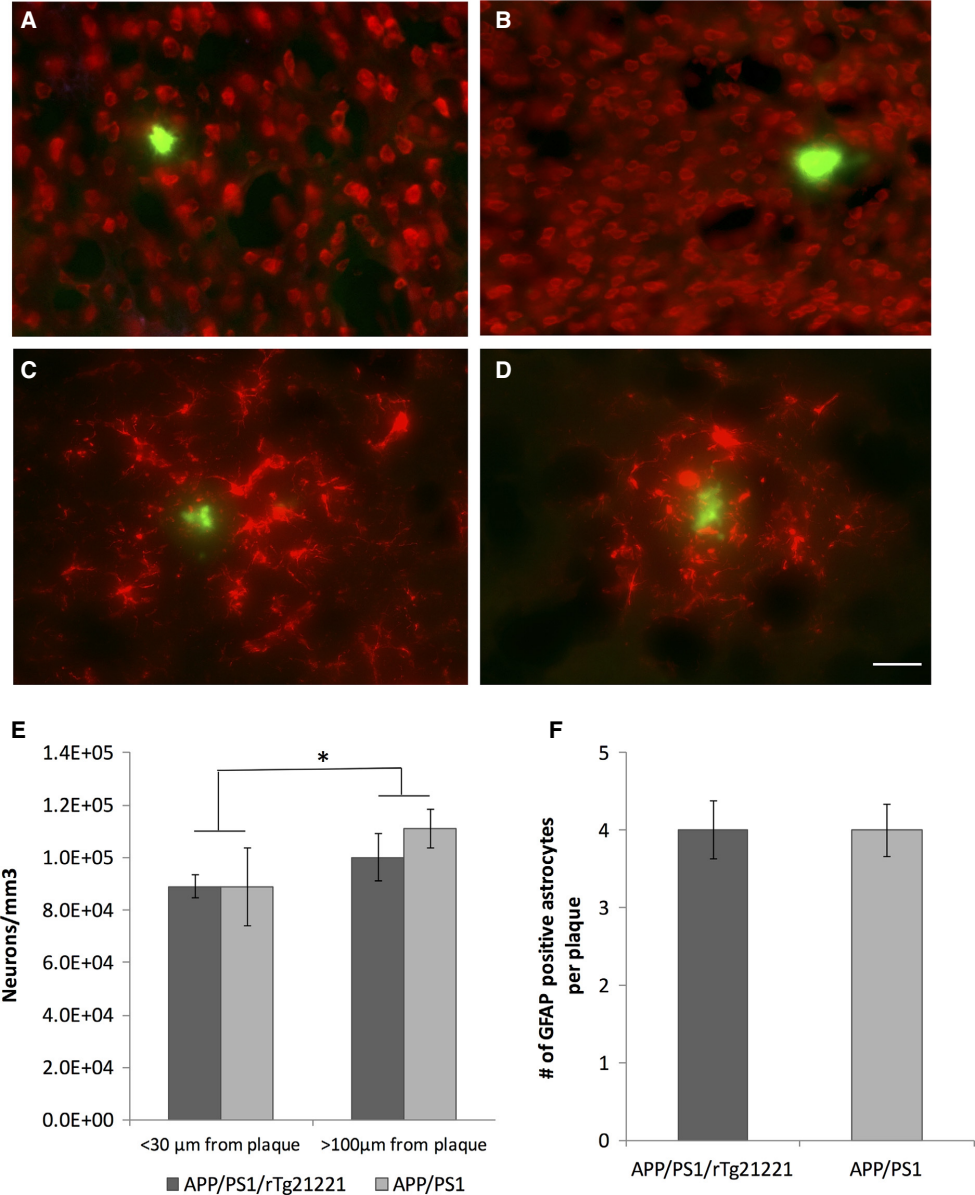
Overexpressing human tau does not affect plaque‐associated neuron loss and astrogliosis in APP/PS1 mice. Representative images of neurons (red, *Neurotrace* stained; A,B) and GFAP‐positive astroglia (red; C,D) and ThioS‐positive plaques (green) in APP/PS1 (A,C) and APP/PS1/rTg21221 (B,D) mice. Neurons were counted in a 30 × 30 μm box for areas near (< 30 μm) and far (> 100 μm) from plaques and as expected there was a decrease in neuronal density in the immediate vicinity of plaques (*two‐way anova *F*_1,12_ = 6.852, *p* = 0.022). No difference was seen between genotypes (E). GFAP‐positive astrocytes counted in a radius of 30 μm around ThioS‐positive plaques showed no difference either (F). APP/PS1/rTg21221 *n* = 5, APP/PS1n = 3, scale bar is 30 μm. [Colour figure can be viewed at wileyonlinelibrary.com].

Neuronal loss is one of the key neuropathological hallmarks of Alzheimer's disease. In plaque‐bearing mice, there is generally not much overt neuronal loss without overexpressing FTD mutant tau. Subtle plaque‐associated neuron loss has been reported for APP/PS1 mice (Rupp *et al*., 2011). We assessed the impact of human tau overexpression on plaque‐associated neuronal loss. As described previously, we observed subtle neuronal loss near plaques (two‐way anova *F*_1,12_ = 6.852, *P* = 0.022 for regions near plaques vs. far from plaques). We did not see any exacerbation of plaque‐associated neuronal loss in APP/PS1/rTg21221 mice (two‐way anova,* P* > 0.05 for genotype and genotype × plaque distance interaction, Fig. 2).

Plaque deposition is associated with local gliosis and degenerative changes in neurites including dystrophic swellings that accumulate pathological forms of tau (McLellan *et al*., 2003; Spires *et al*., 2005). To examine whether human tau overexpression affects gliosis, activated astrocytes (GFAP‐positive) within 30 μm of a plaque were counted in APP/PS1/rTg21221 and APP/PS1 mice (Fig. 2). No change in the number of activated astrocytes per plaque was detected (Student *T*‐Test *P* > 0.05), and quantitative western blot of cortical homogenates confirmed no global change in the amount of GFAP (Fig. S1).

**Figure 3:**
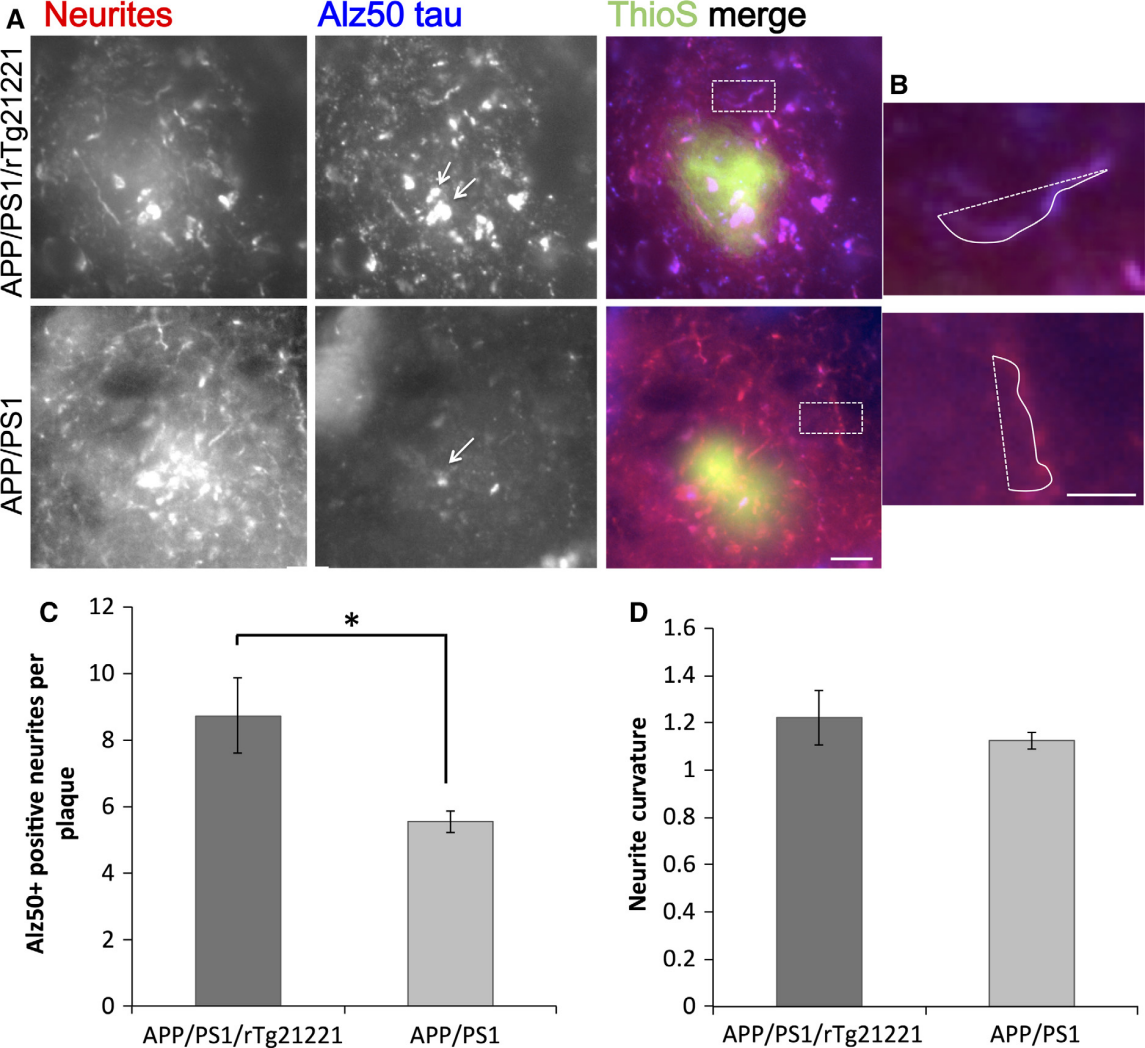
Overexpression of human tau exacerbates plaque‐associated dystrophic neurites but does not affect neurite curvature. Staining of cortical sections neurites (smi312, red), misfolded tau (Alz50, blue), and plaques (ThioS, green) shows the accumulation of tau‐positive dystrophic neurites and the abnormal curvature of neurites near plaques in both APP/PS1 and APP/PS1/rTg21221 mice (A). Neurite curvature was measured by dividing the length (solid line) by the end‐to‐end distance (dotted line) of each neurite segment (B). Quantification reveals an increase in Alz50‐positive dystrophic neurites around plaques in APP/PS1/rTg21221 mice (C) (*t‐test df = 0.047, *P* = 0.047), whereas the neurite curvature does not change in presence of human tau (D). APP/PS1/rTg21221 *n* = 5, APP/PS1 *n* = 3, scale bars are 10 μm (A) and 3 μm (B). [Colour figure can be viewed at wileyonlinelibrary.com].

**Figure 4:**
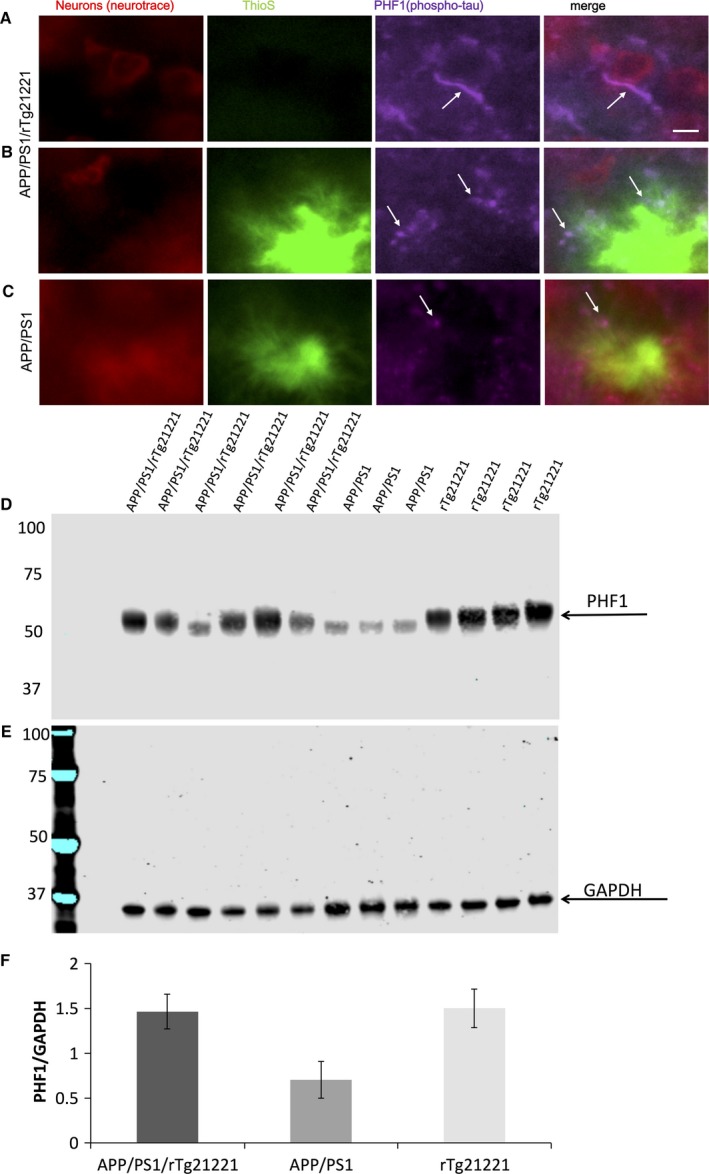
Overexpression of human tau does not cause tangle formation or increase tau hyperphosphorylation. PHF1 positive tau accumulates in neuropil threads in APP/PS1/rTg21221 mice (A) and in dystrophic neurites in both APP/PS1/rTg21221 (B) and APP/PS1 mice (C). A western blot of crude homogenate from the cortex of a mouse (5 μg protein) was probed for PHF1 (D) and GAPDH (E) as a loading control. The PHF1 bands between 55–65 kDa were quantified and the overexpression of human tau did not change the overall levels of PHF1 (F). APP/PS1/rTg21221 *n* = 6, APP/PS1n = 3, rTg21221 *n* = 4 Scale bar 5 μm. [Colour figure can be viewed at wileyonlinelibrary.com].

Staining of brain sections with ThioS, PHF1 and Alz50 did not show neurofibrillary tangles for either genotype (Fig. 1 for ThioS, Fig. 3 for Alz50 and Fig. 4 for PHF1;). However, Alz50‐positive and PHF1‐positive tau accumulations were observed in dystrophic neurites around plaques (Figs 3 and 4). To examine the toxic effect of Aβ and hTau on neurites around plaques, brain sections were co‐immunostained for misfolded tau (Alz50) and neurofilaments (smi312), and dystrophic Alz50‐positive neurite swellings (diameter > 2.5 μm) associated plaques were counted (Fig. 3A). With overexpression of human tau, there was a significant increase in dystrophies per plaque (df = 6, *P* = 0.036 Student's *T*‐Test, Fig. 3C). Curvature of smi312‐positive neurites, which is known to increase around Aβ‐plaques (Garcia‐Alloza *et al*., 2006a), was not significantly altered in APP/PS1/rTg21221 compared to APP/PS1 mice (*P* > 0.05 Mann–Whitney *U* test, Fig. 3D). PHF1 staining also showed an absence of tangles but the presence of dystrophic neurites and neuropil threads in APP/PS1/rTg21221 mice. APP/PS1 mice without human tau also demonstrated plaque‐associated PHF1‐positive neuritic dystrophies but qualitatively less neuropil threads far from plaques (Fig. 4). Western blot analysis indicated that there is a trend toward change (anova *F*_2,12_ = 3.614, *P* = 0.066) in the amount of phosphorylated tau in APP/PS1/rTG21221 compared with APP/PS1 alone or when compared with rTG21221 mice with no APP/PS1 (Fig. 4).

Together, these histologic data indicate an increase in amyloid deposition and exacerbation of neuritic dystrophies around plaques with the overexpression of human tau in APP/PS1 mice; in the absence of neurofibrillary tangle pathology.

### No effect of hTau overexpression on Aβ‐mediated synaptic loss

**Figure 5:**
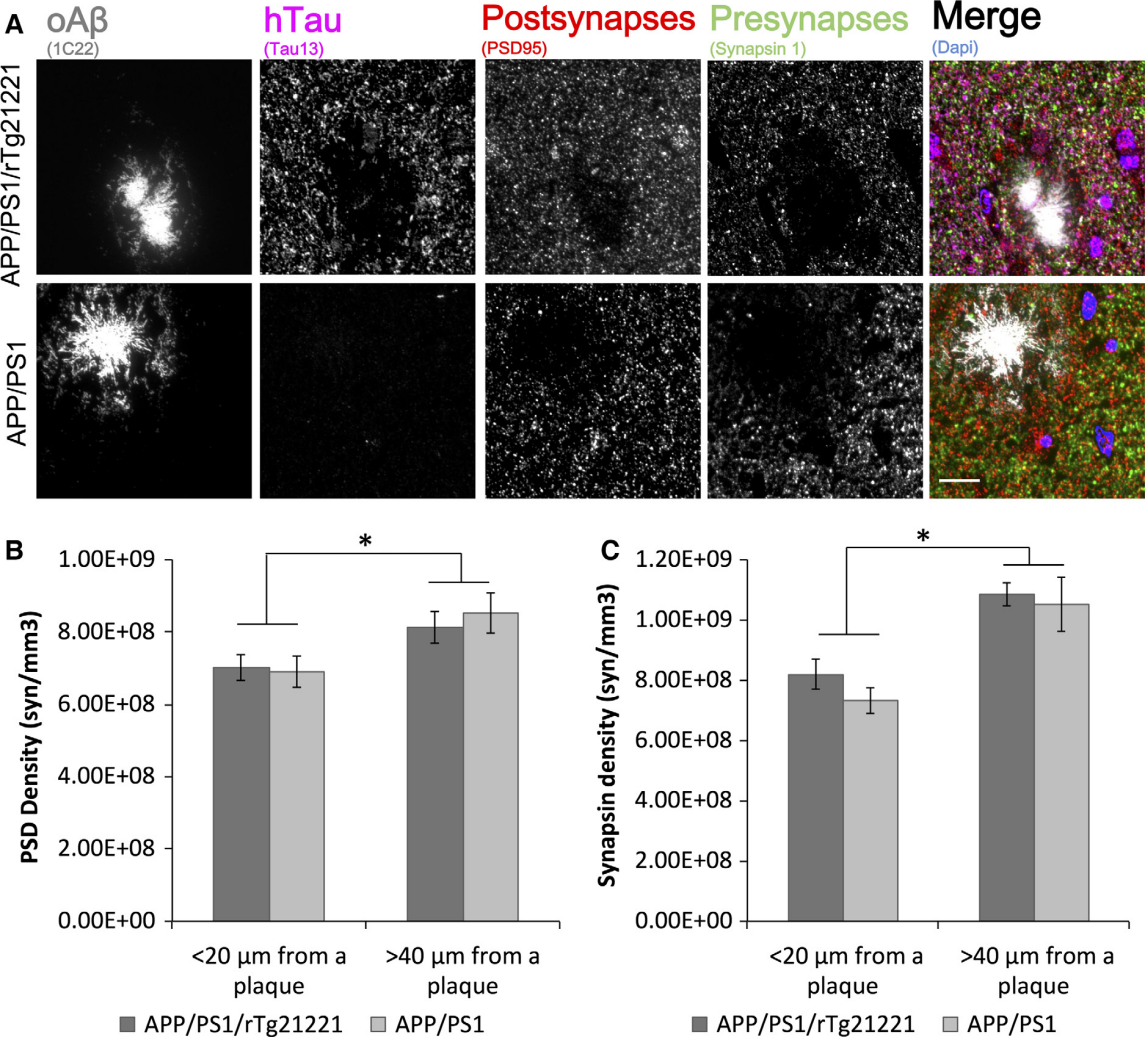
Overexpression of human tau does not increase synapse loss in APP/PS1 mice. To investigate synapse loss, array tomography ribbons from APP/PS1 (*n* = 6) and APP/PS1/rTg21221 mice (*n* = 5) were stained for oligomeric Aβ (oAβ; 1C22), human tau (Tau13), post‐synapses (PSD95), and pre‐synapses (synapsin‐1) (A). There was synapse loss in both genotypes within 20 μm of plaques (*PSD data effect of plaque distance *F*_1,21_ = 8.4, *P* = 0.01; synapsin data *F_1.21_ = 16.6, *P* = 0.001). There is no exacerbation of synapse loss with expression of human tau of either post‐synaptic terminals (B) or pre‐synaptic terminals (C) (two‐way anova effect of genotype *F* < 0.5, *P* > 0.05). Scale bar is 10 μm. [Colour figure can be viewed at wileyonlinelibrary.com].

To determine if the overexpression of wild‐type human tau increased Aβ‐induced synaptic loss, array tomography was used to quantify synapse density in the neocortex. Tissue ribbons were stained for oligomeric Aβ using the conformer‐specific 1C22 antibody. A total of 126 210 synapses in APP/PS1 (*n* = 6 animals) and 139 412 synapses in APP/PS1/rTg21221 mice (*n* = 5 animals) were analyzed. No change in synapse density was seen for either the pre‐synaptic marker synapsin‐1 or the post‐synaptic marker PSD95 (Fig. 5). However, both APP/PS1 and APP/PS1/rTg21221 mice showed a significant decrease in the densities of pre‐ and post‐synapses near plaques (< 20 μm from plaque border) compared to distant from plaques (> 40 μm from plaque border), similar to what has been reported previously (Koffie *et al*., 2009). Western blot analysis of the pre‐synaptic marker synaptophysin in crude cortical homogenates also suggested no change in synaptic protein levels between APP/PS1 and APP/PS1/rTg21221 mice (Fig. S2).

**Figure 6:**
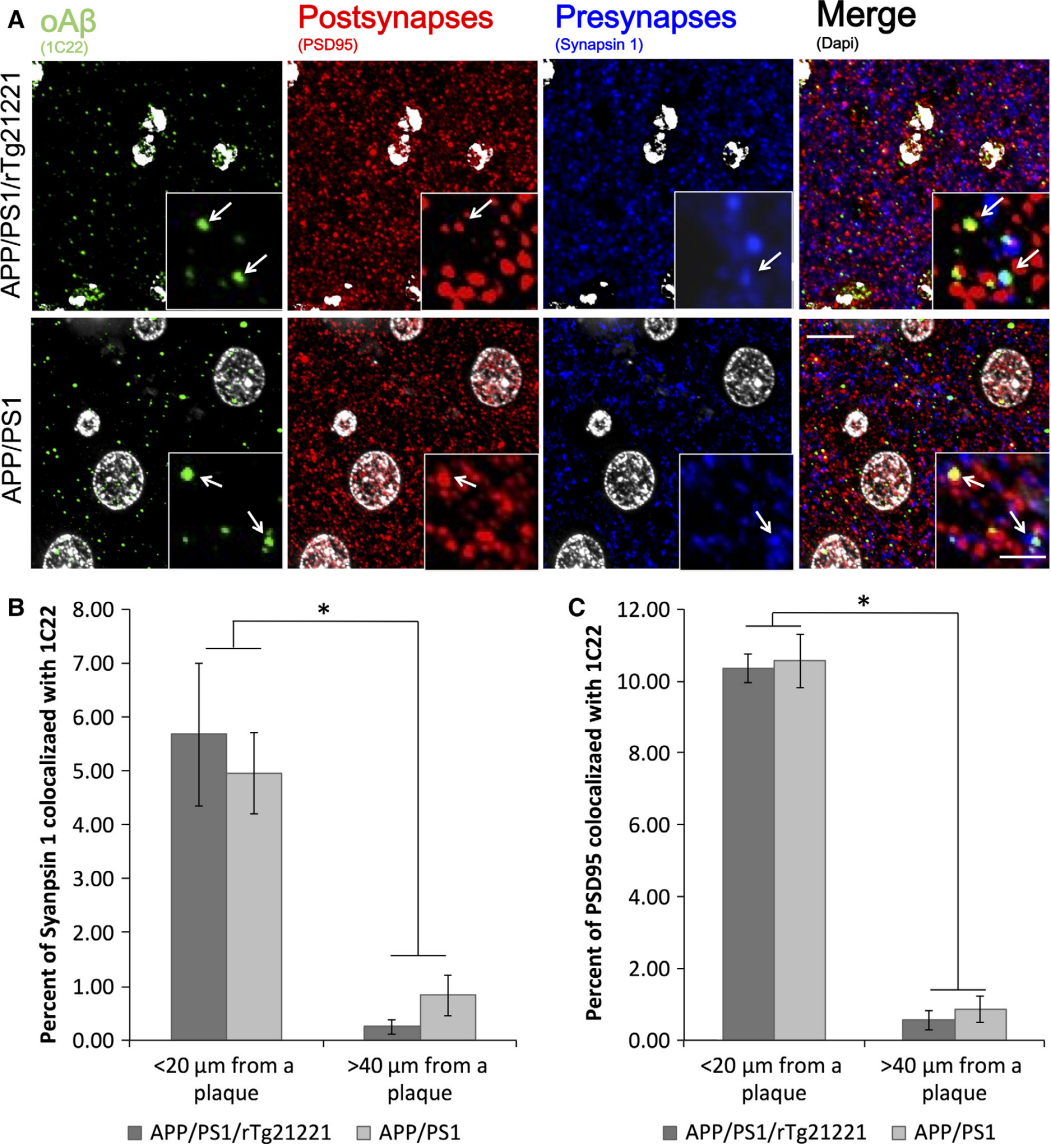
Overexpression of human tau does not affect the localization of Aβ at synapses. Analysis of the co‐localization of Aβ labeled with 1C22 and PSD95 or synapsin‐1 (A) show that Aβ presence at pre‐synapses (B) or post‐synapses (C) does not change when human tau is overexpressed in APP/PS1/rTg21221 mice (two‐way anova effect of genotype *F* < 0.3, *P* > 0.05). In both pre and post synapses, there is a significantly higher percentage of synapses containing Aβ near plaques vs. far from plaques (PSD data effect of plaque distance *F_1,21_ = 363.6, *P* = 2.19 × 10^−13^; synapsin data *F_1,21_ = 13.3, *P* = 0.002). APP/PS1/rTg21221 *n* = 5, APP/PS1 *n* = 6, scale bar is 10 μm, scale bar for insert is 2 μm. [Colour figure can be viewed at wileyonlinelibrary.com].

As there is evidence that the accumulation of Aβ at synapses contributes to synaptic shrinkage and loss (Koffie *et al*., 2009), we next assessed the co‐localization of synaptic markers with the oligomeric Aβ antibody 1C22 (Fig. 6). While both genotypes had significantly more Aβ at synapse near plaques than far from plaques, the overexpression of human tau did not increase the amount of Aβ found co‐localized with the synapse. This finding was confirmed biochemically by assaying synaptoneurosome preparations using a human Aβ ELISA (Fig. S3A), and by western blot analysis of the same preparations using Aβ antibody (82E1; Fig. S3B and C); both assays showed no difference between mice APP/PS1 and APP/PS1/rTg21221 mice. Furthermore, the presence of Aβ in APP/PS1/rTg21221 mice did not increase the amount of human tau found in synaptoneurosomes compared to rTg21221 mice (Fig. S3D and E).

## Discussion

From genetic data, it is clear that changes in APP processing, leading to increased Aβ42 levels, initiate the disease process in familial AD (fAD), and likely also in sporadic AD (Hardy & Selkoe, 2002). It is also clear that pathological changes in tau correlate better than plaque deposition with neuronal death observed in AD (Gomez‐Isla *et al*., 1997). However, the connection between Aβ and tau pathologies remains enigmatic and it is still unknown how changes in amyloid processing can cause neurotoxicity related to tau.

The synergistic effects of Aβ and tau in the deposition of the classic pathological lesions – plaques and neurofibrillary tangles – have been modeled in mice expressing fAD mutant APP and FTD mutant tau, such as the 3×Tg line (Oddo *et al*., 2003), which develops both pathologies. In these mice, amyloid deposition precedes tau deposition, and the removal of Aβ by immunotherapy also reduces early tau pathology (phospho‐tau) but tangles remain unchanged (Oddo *et al*., 2004). However, immunotherapies directed against tau do not affect Aβ, indicating a role for Aβ upstream of tau pathology (Walls *et al*., 2014). Recently, we generated mouse model that expresses brain‐wide human mutant APP and human mutant P301L tau only in the entorhinal cortex by crossing APP/PS1 mice with rTgTauEC mice (Pooler *et al*., 2015). In these mice, the presence of human Aβ accelerates tau propagation through the brain and increases plaque size and plaque‐associated dystrophic neurites. Together, these studies support the idea of an interaction between plaques and tangles, at least in the presence of disease‐associated mutations in both Aβ and tau. In contrast, at the relatively young age we examined in our focus on synapse loss, we did not observe neurofibrillary tangles in APP/PS1 mice overexpressing wild‐type human tau (APP/PS1/rTg21221), however, we found larger plaques and exacerbated dystrophic neurites, similar to our previous observations in APP/PS1xrTgTauEC mice (Pooler *et al*., 2015). A recent study found that mice with high levels of human Aβ oligomers do develop neurofibrillary tangle pathology at much older ages (18 months) when crossed with wild‐type human tau expressing mice (Umeda *et al*., 2014). This is likely due to the different ages examined (as here we focus on earlier synaptic changes) but could also be due to the different forms of APP and tau expressed in the two lines.

Synapse loss is the strongest pathological correlate of dementia (DeKosky & Scheff, 1990; Terry *et al*., 1991), and is thought to be the key pathogenic process driving AD symptoms (Spires‐Jones & Hyman, 2014). Synaptic degeneration downstream of oligomeric Aβ has been very well established not only in animal models of the disease (Walsh *et al*., 2002), but also in several studies of human brains (Koffie *et al*., 2012; Perez‐Nievas *et al*., 2013; Bilousova *et al*., 2016). Animal models of FTD expressing mutant human tau also exhibit synaptic loss and dysfunction, along with pronounced neurodegeneration (Rocher *et al*., 2010; Crimins *et al*., 2013; Menkes‐Caspi *et al*., 2015). From these data, it became clear that pathological changes in both can independently drive synapse loss, but it is unclear whether Aβ and tau act on the same pathway to synapse degeneration.

When plaque‐bearing mice are crossed onto a mouse tau knock‐out strain (*Mapt* ^*0/0*^), the Aβ‐induced synaptic phenotypes – including seizures and LTP deficits – become ameliorated. The removal of tau was also found to be protective against memory loss associated with Aβ expression in mice, likely due to protection against synapse loss (Roberson *et al*., 2007, 2011; Shipton *et al*., 2011). These findings provided evidence for the synergistic action of Aβ and tau toward synapse dysfunction. Here, we directly tested whether overexpression of wild‐type human tau exacerbates synaptic loss associated with Aβ‐plaques. Surprisingly, the increased level of non‐mutant tau in APP/PS1/rTg21221 mice did not worsen the synapse pathology related to Aβ. Together with the *Mapt* ^*0/0*^ data, this indicates that the endogenous mouse tau is sufficient to cause the negative effects associated with Aβ, and that there is a ceiling effect of the requirement of tau for Aβ‐mediated synapse toxicity. Indeed, it has been shown that the knock‐out of endogenous mouse tau reduces the neurotoxicity of overexpressed human mutant P301L tau (Wegmann *et al*., 2015). However, given that in human brain‐derived synaptosomes, a recent study observed that phospho‐tau is increased in Aβ‐positive synaptosomes in early AD (Bilousova *et al*., 2016), it is possible that at the time point studied here, synapse loss had already reached its maximum level and thus it was too late to see the potentially early effects of human tau expression. Together, these data indicate that endogenous tau may play an important role in the neurotoxicity of tau and Aβ in mouse models and that this role may be at earlier stages of the disease. As the data do not refute the possibility that tau and Aβ are on different pathways to synapse loss, a mouse model that expressed wild‐type human tau in the absence of mouse tau studied at multiple time points would be beneficial to studying the possible interaction between these two important molecules in the context of human AD and synapse loss.

## Author contributions

Performed experiments – RJJ, NR, AGH, SC, JSK, VP, JJRR, RP, SW, MG‐A. Experimental design and analysis – RJJ, NR, GAC, BTH, TS‐J. Wrote and commented on manuscript – RJJ, SW, GAC, MG‐A, BTH, TS‐J.

## Conflict of interests

All authors declare no competing interests.

## Supporting information

Fig. S1. Overexpression of human tau does not affect reactive astrocyte protein levels. A western blot of crude homogenate from the cortex of a mouse (5 μg protein) was probed for GFAP (A) and GAPDH (B) as a loading control. The GFAP band at 55 kDa was quantified and the overexpression of human tau did not change the overall levels of GFAP (C). APP/PS1/rTg21221 *n* = 5, APP/PS1n = 3.

Fig. S2. Overexpression of human tau does affect synapse protein levels. Western blot of crude homogenates from mouse cortices (5 μg protein) probed for (A) synaptophysin and (B) α‐tubulin as a loading control. The overexpression of human tau in APP/PS1 mice did not change the levels of synaptophysin (C). APP/PS1/rTg21221 *n* = 5, APP/PS1 *n* = 4, rTg21221 *n* = 5.

Fig. S3. Overexpression of human tau does not affect protein levels at the synapse. ELISA of synaptoneurosomes showed no difference in Aβ42 levels between APP/PS1 and APP/PS1/rTg21221 (A) Western blot of synaptoneurosomes (5 μg protein) was probed for (B) Aβ (82E1) and (C) human tau (tau13) with β‐actin as loading control. The overexpression of human tau did not change the amount of Aβ found in synaptoneurosome when comparing APP/PS1/rTg21221 with APP/PS1 mice (D). Furthermore, Aβ did not affect the amount of human tau found in the synaptoneurosome when comparing APP/PS1/rTg21221 with rTg21221 mice (E). APP/PS1/rTg21221 *n* = 5, APP/PS1 *n* = 3, rTg21221 *n* = 4.
