# Supplementary figures and images for: Human tau increases amyloid β plaque size but not amyloid β‐mediated synapse loss in a novel mouse model of Alzheimer's disease
Source: Eur J Neurosci. 2016 Nov 12;44(12):3056–66. doi: 10.1111/ejn.13442 (PMC5215483; doi:10.1111/ejn.13442)

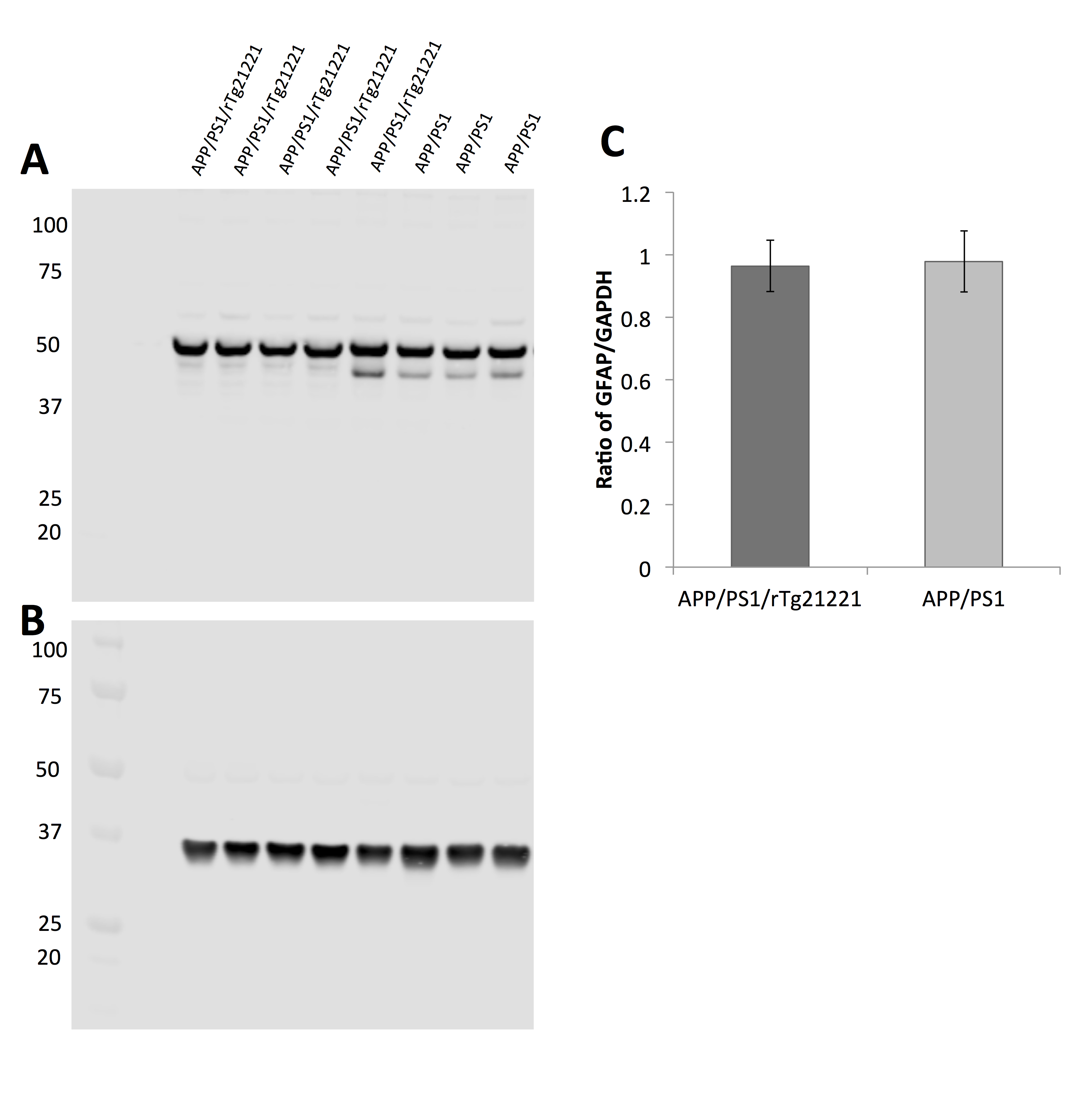

Supplement: Supplementary file 1 — Fig. S1. Overexpression of human tau does not affect reactive astrocyte protein levels. A western blot of crude homogenate from the cortex of a mouse (5 μg protein) was probed for GFAP (A) and GAPDH (B) as a loading control. The GFAP band at 55 kDa was quantified and the overexpression of human tau did not change the overall levels of GFAP (C). APP/PS1/rTg21221 n = 5, APP/PS1n = 3. [file EJN-44-3056-s001.tiff]

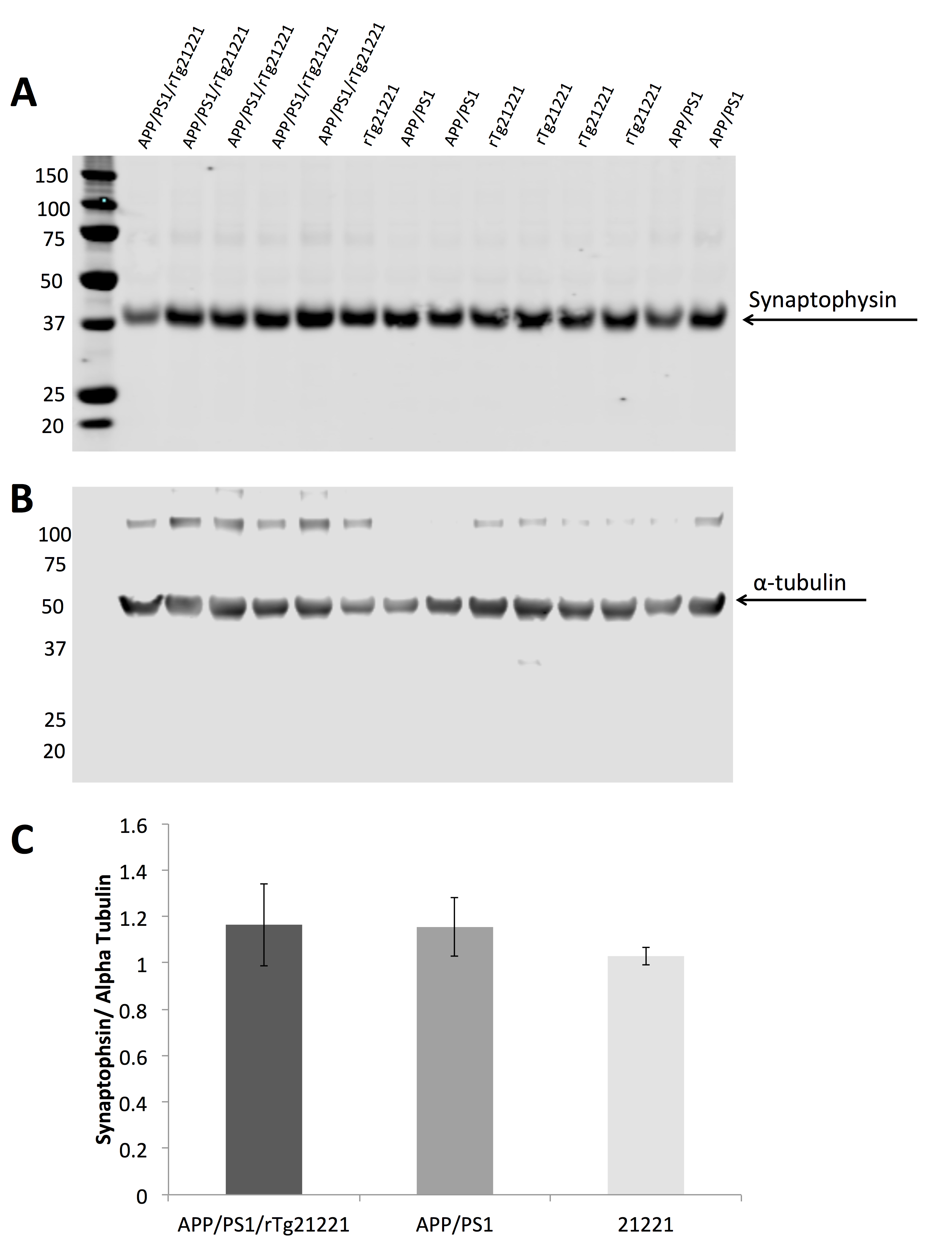

Supplement: Supplementary file 2 — Fig. S2. Overexpression of human tau does affect synapse protein levels. Western blot of crude homogenates from mouse cortices (5 μg protein) probed for (A) synaptophysin and (B) α‐tubulin as a loading control. The overexpression of human tau in APP/PS1 mice did not change the levels of synaptophysin (C). APP/PS1/rTg21221 n = 5, APP/PS1 n = 4, rTg21221 n = 5. [file EJN-44-3056-s002.tiff]

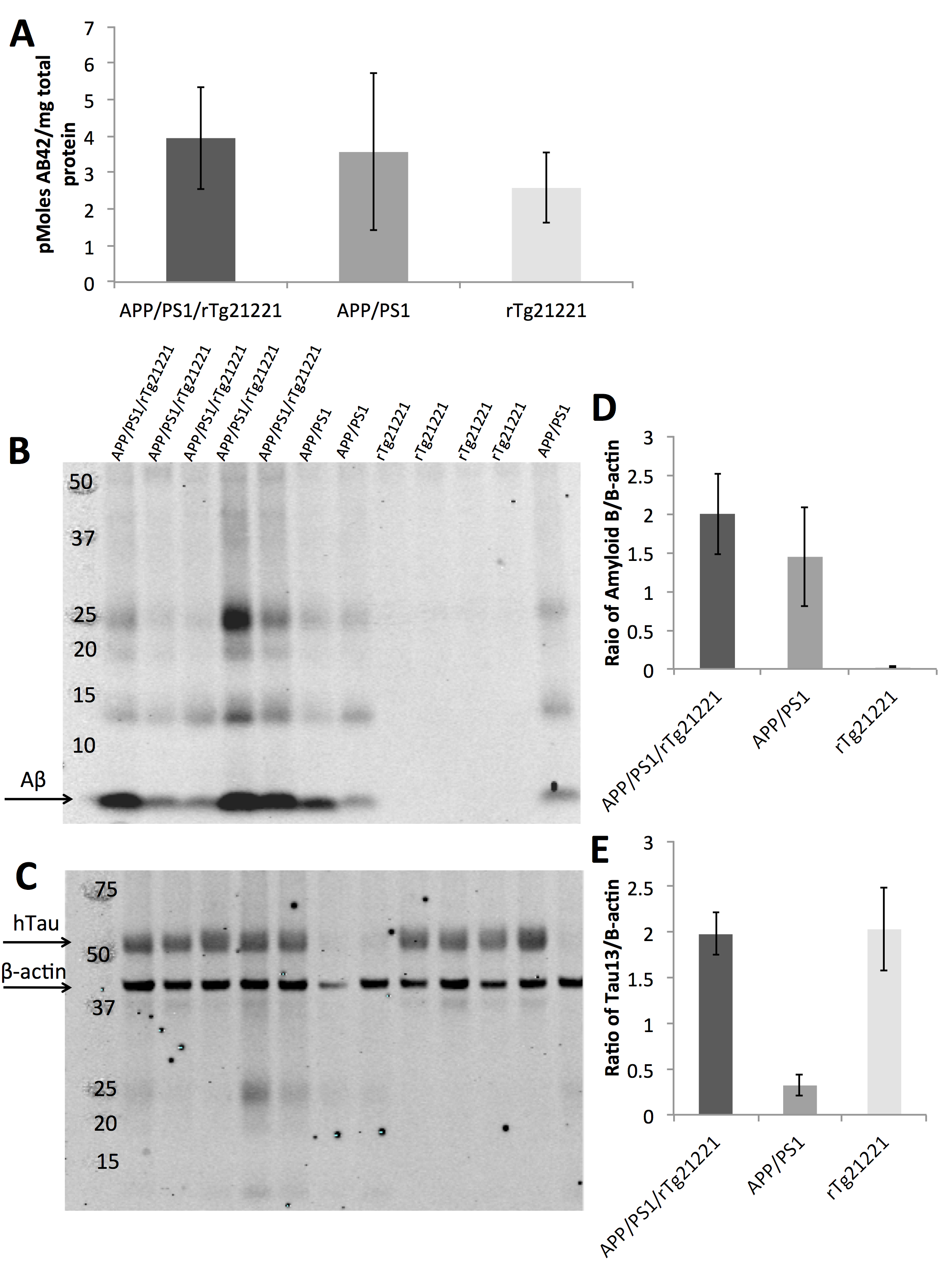

Supplement: Supplementary file 3 — Fig. S3. Overexpression of human tau does not affect protein levels at the synapse. ELISA of synaptoneurosomes showed no difference in Aβ42 levels between APP/PS1 and APP/PS1/rTg21221 (A) Western blot of synaptoneurosomes (5 μg protein) was probed for (B) Aβ (82E1) and (C) human tau (tau13) with β‐actin as loading control. The overexpression of human tau did not change the amount of Aβ found in synaptoneurosome when comparing APP/PS1/rTg21221 with APP/PS1 mice (D). Furthermore, Aβ did not affect the amount of human tau found in the synaptoneurosome when comparing APP/PS1/rTg21221 with rTg21221 mice (E). APP/PS1/rTg21221 n = 5, APP/PS1 n = 3, rTg21221 n = 4. [file EJN-44-3056-s003.tiff]
