# Supplementary material for: Human tau increases amyloid β plaque size but not amyloid β‐mediated synapse loss in a novel mouse model of Alzheimer's disease
Source: Eur J Neurosci. 2016 Nov 12;44(12):3056–66. doi: 10.1111/ejn.13442 (PMC5215483; doi:10.1111/ejn.13442)
Supplement: Supplementary file 4 [file EJN-44-3056-s004.pdf]

Supplemental Figure 1: Overexpression of human tau does not affect reactive astrocyte protein levels. A western blot of crude homogenate from the cortex of a mouse (5 µg protein) was probed for GFAP (A) and GAPDH (B) as a loading control. The GFAP band at 55kDa was quantified and the overexpression of human tau did not change the overall levels of GFAP (C). APP/PS1/rTg21221 n=5, APP/PS1n=3

Supplemental Figure 2: Overexpression of human tau does affect synapse protein levels. Western blot of crude homogenates from mouse cortices (5 µg protein) probed for (A) synaptophysin and (B) α-tubulin as a loading control. The overexpression of human tau in APP/PS1 mice did not change the levels of synaptophysin (C). APP/PS1/rTg21221 n=5, APP/PS1 n=4, rTg21221 n=5

Supplemental Figure 3: Overexpression of human tau does not affect protein levels at the synapse. ELISA of synaptoneurosomes showed no difference in Aβ42 levels between APP/PS1 and APP/PS1/rTg21221 (A) Western blot of synaptoneurosomes (5 µg protein) was probed for (B) Aβ (82E1) and (C) human tau (tau13) with β-actin as loading control. The overexpression of human tau did not change the amount of Aβ found in synaptoneurosome when comparing APP/PS1/rTg21221 with APP/PS1 mice (D). Furthermore, Aβ did not affect the amount of human tau found in the synaptoneurosome when comparing APP/PS1/rTg21221 with rTg21221 mice (E). APP/PS1/rTg21221 n=5, APP/PS1 n=3, rTg21221 n=4.
